# Will histone deacetylase inhibitors require combination with other agents to fulfil their therapeutic potential?

**DOI:** 10.1038/sj.bjc.6604557

**Published:** 2008-08-19

**Authors:** L Nolan, P W M Johnson, A Ganesan, G Packham, S J Crabb

**Affiliations:** 1 Cancer Research UK Clinical Centre, School of Medicine, University of Southampton, Southampton General Hospital, Southampton, SO16 6YD, UK; 2 School of Chemistry, University of Southampton, Southampton General Hospital, Southampton, SO16 6YD, UK

**Keywords:** histone deacetylase, inhibitor, combination therapy

## Abstract

Histone deacetylase inhibitors have progressed rapidly from the laboratory to clinical testing. This review highlights the promising data for their combination with a wide range of established and novel anticancer agents and discusses the mechanisms that underpin these interactions.

Histone deacetylase inhibitors (HDIs) have progressed rapidly from the laboratory to clinical testing as novel anticancer agents, culminating in the approval of SAHA (Vorinostat, Merck, Whitehouse Station, NJ, USA) for the treatment of recurrent cutaneous T-cell lymphoma. However, despite their promising activity in pre-clinical models, HDIs have demonstrated only modest antitumour activity in initial clinical trials in solid malignancies. In this review, we will discuss current findings to support the hypothesis that in most scenarios, combination with other therapeutic modalities will be required to optimise efficacy and current evidence for the molecular mechanisms that underpin potential combinations.

## HDIs – structure and mechanism of action

**Figure 1:**
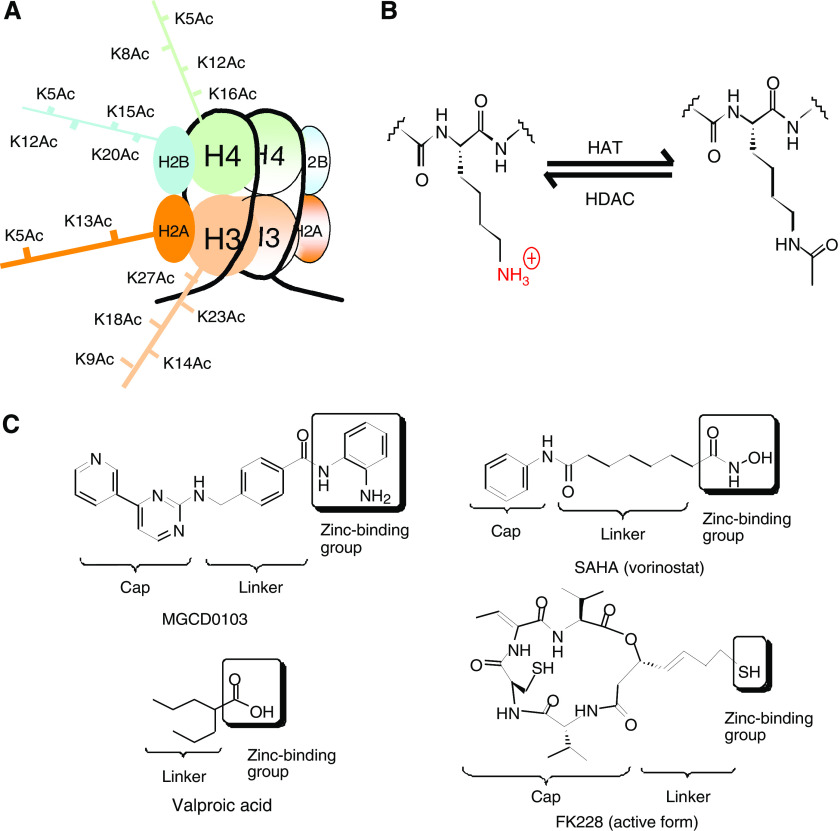
(**A**) Structure of core histone with sites of N-terminal lysine side-chain acetylation shown. (**B**) Histone deacetylase (HDAC) enzymes counter the activity of histone acetyltransferases (HAT) by inducing hydrolysis of the *ε*-amino acetyl moiety on specific acetylated lysine residues. (**C**) Structure of HDIs in clinical development, demonstrating conformation to a common pharmacophore.

Histone deacetylase (HDAC) enzymes counter the activity of histone acetyltransferases by inducing hydrolysis of the *ε*-amino acetyl moiety on specific acetylated lysine residues within core histones (Figure 1A and B). Histone acetylation participates in transcriptional regulation in concert with other epigenetic events such as DNA and histone methylation. Histone deacetylase inhibitors induce accumulation of acetylated histones, resulting in the relaxation of chromatin structure and promoting access to transcriptional machinery. Surprisingly, these transcriptional effects are relatively selective, only affecting around 3–10% of the transcriptome, with both the induction and repression of gene expression targets. There may be a common gene expression signature in response to HDIs. For example, reversal of epigenetic silencing of the p21^WAF1/CIP1^ cyclin-dependent kinase inhibitor is observed in various cancer cell types and for all HDIs. However, cell-specific effects such as modulation of nuclear hormone receptors or cell signalling pathways may vary in biological importance in particular cancer types, which may be relevant for combination strategies.

**Figure 2:**
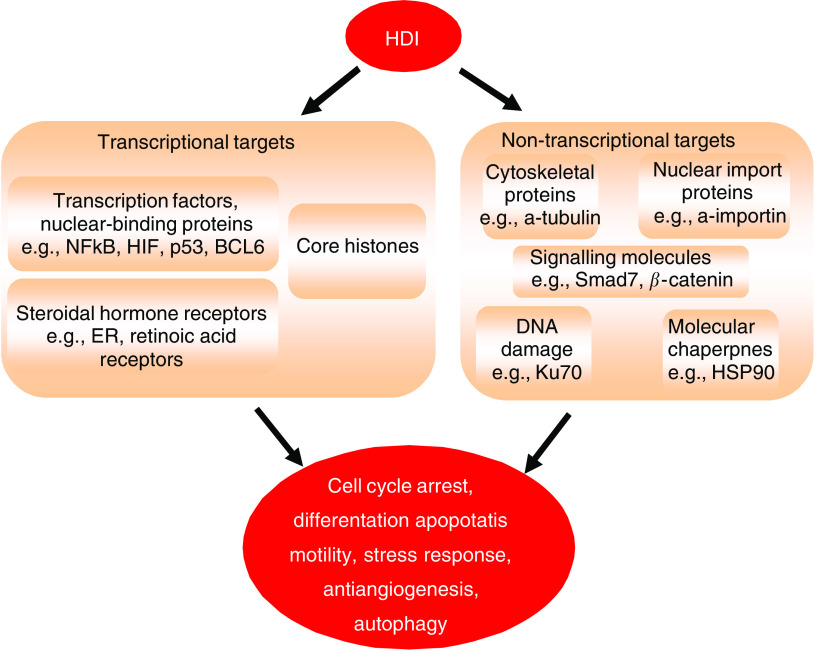
The mechanisms of action of HDIs are varied with the modification of acetylation status of many non-histone proteins as well as core histones, resulting in a wide range of biological effects.

Although the modulation of transcription through histone modification serves as a useful paradigm, the mechanisms that mediate the anticancer effects of HDIs are more complex, with many non-histone targets identified (Figure 2).

There are multiple HDIs in clinical development and key representatives are shown in Figure 1C. These molecules are structurally diverse, but with the common feature of employing a chemical ‘war-head’ active site that chelates the zinc atom in the active site of Class I and/or II HDACs, thereby blocking enzyme activity. With the exception of very simple aliphatic acids such as valproic acid (VPA), these molecules conform to a common pharmacophore (Figure 1C), comprising a ‘linker’ mimicking a lysine side chain, and a ‘cap’ structure of variable size that makes additional interactions around the rim of the enzyme active site.

## HDI combinations with cytotoxic chemotherapy

In an attempt to find a niche for HDIs, they have been tested in combination with a variety of conventional cytotoxic chemotherapeutic agents. Pre-clinical data in multiple cancer cell lines (including breast, ovarian, pancreatic, colon, non-small cell lung, prostate, thyroid, hepatocellular and oral squamous cell carcinomas and melanoma) have shown the potentiation by HDIs of the effects of topoisomerase I inhibitors (camptothecin, irinotecan, topotecan) topoisomerase II inhibitors (epirubicin, doxorubicin, etoposide, mitoxantrone) and other DNA-damaging agents (cisplatin, oxaliplatin, bleomycin). *In vitro*, synergistic induction of apoptosis is seen when HDIs are combined with epirubicin in breast cancer cells and with etoposide or cisplatin in melanoma cells (Marchion *et al*, 2005; Valentini *et al*, 2007). *In vivo* potentiation of epirubicin has been confirmed in breast cancer xenograft models (Marchion *et al*, 2005). Subsequently, a phase I clinical trial of VPA combined with epirubicin showed a 22% partial response rate in multiply pretreated solid malignancies (Munster *et al*, 2007). It is worth noting that these investigators began VPA 48 h before infusion of epirubicin on each cycle to attempt to exploit synergism seen with this sequencing approach demonstrated in pre-clinical models (see below). Dose-limiting toxicity was predominantly neurovestibular (including dizziness, confusion and hearing loss) or gastrointestinal (diarrhoea). Anthracycline-induced toxicity in this trial did not appear to be worsened by combination with VPA, and other toxicities are manageable providing some reassurance that any synergy in terms of efficacy will not be replicated in unwanted side effects. A phase II trial combining VPA with chemotherapy (5FU, epirubicin and cyclophosphamide) is currently recruiting patients with metastatic breast cancer (www.clinicaltrials.gov).

A number of mechanisms may account for the potentiation of DNA-damaging agents by HDIs, reflecting their pleiotropic actions. For topoisomerase inhibitors, HDAC1 and -2 have been shown to bind and interact with topoisomerase II, and form an integral part of the NuRD complex (Tsai *et al*, 2000). Scheduling appears to be critical in explaining the variable potentiating effects of HDIs on topoisomerase II inhibitors, with pre-exposure of breast cancer cells to vorinostat for 48 h needed to induce synergistic apoptosis, increase nuclear epirubicin levels and increase DNA damage. Shorter pre-exposure periods abrogated synergy, and exposure after chemotherapy resulted in antagonistic effects, implying that HDI relaxation of chromatin allows greater access for topoisomerase II inhibition, but possibly also stabilises the topoisomerase II–DNA complex further resulting in more efficient generation of strand breaks. Expression of the chemotherapeutic target may also be crucial as potentiation was lost in topoisomerase II null cells if the HDI was combined with epirubicin but not topotecan (Marchion *et al*, 2004).

Sequencing was also important for combination with topoisomerase I inhibition but with apparent benefit for HDI exposure after the chemotherapeutic to exploit cell cycle effects of each agent. Potentiation has been shown for an HDI added 24–48 h after camptothecin in breast and lung cancer cells. Cells arrested in G2-M by camptothecin appeared most sensitive to subsequent HDI addition possibly through HDI-induced decreases in cyclin B levels and of the antiapoptotic proteins XIAP and survivin. These findings suggested that reduced expression of these antiapoptotic factors could increase efficacy of topoisomerase I inhibitors if given in a sequence that does not prevent tumour cell progression through S phase (Bevins and Zimmer, 2005). Enhancement of cisplatin-induced apoptosis by HDIs in oral squamous cell carcinoma has also been shown to be greater if the HDI is given concurrently or following chemotherapy rather than prior. Experiments suggested that cells arrested at the G1/S checkpoint by cisplatin were more sensitive to HDAC inhibition through enhancement of reactive oxygen species generation and caspase-3 activation. Histone deacetylase inhibitor therapy decreased intracellular reduced glutathione. Thus, HDIs appeared to disrupt intracellular redox balance, inducing maximal apoptosis at G1/S arrest and potentiating platinum response (Sato *et al*, 2006). Taken together, there is mounting pre-clinical evidence that HDIs synergistically potentiate chemotherapeutic agents that exploit topoisomerase enzymes and DNA damage. Clinical investigation now in progress will elucidate whether these promising findings translate to the clinic and also if interactions impact on toxicity (Table 1).

Taxanes, which inhibit microtubule depolymerisation during metaphase resulting in increased microtubule formation and activation of mitosis checkpoints leading to apoptosis, have also been investigated in combination with HDIs. Synergistic reductions in growth were seen in endometrial cancer cells following treatment with paclitaxel combined with the HDI trichostatin A (TSA), and this was confirmed in mouse xenograft studies (Dowdy *et al*, 2006). Synergistic interaction was also seen in breast cancer cells combining docetaxel with vorinostat (Bali *et al*, 2005). There are no published clinical data for HDI–taxane combinations but trials are ongoing in breast and gynaecologic cancers (Table 1). With regard to underlying mechanisms, in endometrial cancer cell lines, TSA administration induced *α*-tubulin acetylation and appeared to stabilise microtubules. Combination with paclitaxel led to a significant increase in acetylated tubulin and microtubule stabilisation above that with either agent alone (Dowdy *et al*, 2006).

These data show a clear rationale for combining HDIs with a range of chemotherapeutic agents, but that clinical trials must be underpinned by a clear mechanistic rationale specific to both experimental agents and tumour types.

## HDI combinations with agents targeting the human epidermal growth factor receptor (HER) family

Targeted therapies aimed at the HER family have advanced treatment of a range of common malignancies including breast, colorectal and lung cancers. The main data regarding HDI combinations to optimise this approach are for HER2-overexpressing breast cancer. Trastuzumab, a humanised monoclonal antibody to the HER2 extra cellular domain, is effective for those with receptor overexpression; however, optimisation of HER2-targeted therapy and avoidance of resistance mechanisms are required (Crabb and Chia, 2007). *In vitro* studies indicate that HDIs have single-agent activity in HER2-overexpressing breast cancer cell lines including attenuation of HER2 expression, its tyrosine kinase activity, its cell membrane localisation and dimerisation with HER3 (Fuino *et al*, 2003; Bali *et al*, 2005). Combination with trastuzumab produced synergistic induction of apoptosis (Fuino *et al*, 2003; Bali *et al*, 2005). Synergy may result from counteracting HER2 overexpression as HDAC inhibition reduced HER2 mRNA transcript expression and induced HER2 protein degradation (Scott *et al*, 2002; Fuino *et al*, 2003). The latter mechanism occurred through the acetylation of heat shock protein (HSP)90, causing its inactivation and loss of multiple HSP90 client proteins. HSP90 acetylation decreased ATP binding, inducing a shift from HER2 binding with HSP90 to HSP70, resulting in HER2 targeting for ubiquitination and proteasomal degradation (Fuino *et al*, 2003). On the basis of these *in vitro* data, clinical trials of trastuzumab–HDI combinations are in progress for locally advanced and metastatic breast cancer.

It remains unproven to what degree synergism between HDIs and trastuzumab in HER2-positive breast cancer models might also apply to other HER2-directed therapeutics that are in various stages of clinical testing (Crabb and Chia, 2007). However, inhibition of proliferation, apoptosis and signalling inhibition were potentiated when vorinostat was co-administered with the pan-HER tyrosine kinase inhibitor CI-1033 in breast as well as prostate and head and neck squamous carcinoma cells.

Regarding other members of the HER family, in non-small cell lung cancer (NSCLC), synergy has been shown between HDIs and the HER1 (EGFR) tyrosine kinase inhibitors erlotinib and gefitinib (Witta *et al*, 2006). Histone deacetylases are recruited by transcriptional repressors such as Slug/Snail and ZEB1, which are implicated in resistance mechanisms to these agents, and gefitinib sensitivity appeared to be restored in NSCLC cell line models of gefitinib resistance when combined with an HDI (Witta *et al*, 2006). In a separate study, HSP90 acetylation and reduced association with HER1, Akt and STAT3 were seen in cell lines harbouring HER1 kinase mutations following exposure to HDIs leading to apoptosis. Conversely, little effect on apoptosis was seen in cells not dependent on HER1 through kinase mutations. Therefore, HER1 mutation status might be a predictive factor for HDI combination with HER1 inhibitors in this setting. This highlights the value in dissecting the mechanisms underlying beneficial combinations to allow for rational targeting of appropriate cancer phenotypes early in clinical development.

## HDI combination with proteasome inhibition

Proteasome inhibitors act by binding within the catalytic 20S core of the proteasome, resulting in the build-up of proteins targeted for degradation. Cancer cells appear more likely to accumulate misfolded proteins than normal cells, producing a therapeutic window with promising activity in haematological malignancies. Other evidences exist regarding synergistic interactions with HDIs. Treating myeloma cell lines with bortezomib followed by vorinostat produced synergistic induction of mitochondrial injury, caspase activation and apoptosis associated with NF-*κ*B inactivation (Pei *et al*, 2004). Similar findings were also observed in BCR/ABL-positive and -negative leukaemia cell lines and, interestingly, in solid tumour cell lines.

Combined HDAC–proteasomal inhibition may be effective because both interact with NF-*κ*B pathways. Proteasome inhibitors cause accumulation of I*κ*B*α* increasing its NF-*κ*B binding, thereby preventing nuclear localisation and activation of NF-*κ*B target genes. NF-*κ*B subunits are acetylated at multiple lysine residues by p300/CBP acetyltransferases. Acetylation of different residues regulates different NF-*κ*B functions (including transcriptional activation, DNA-binding affinity, I*κ*B*α* assembly and subcellular localisation), and HDIs manipulate gene expression patterns resulting from NF-*κ*B activation both directly through NF-*κ*B subunit acetylation and indirectly through chromatin remodelling (Chen and Greene, 2003; Graham and Gibson, 2005).

## HDI combination with hormonal therapy

Another combination approach for targeting aberrant gene silencing directly is with retinoic acid in acute promyelocytic leukaemia. Acute promyelocytic leukaemia is characterised by translocations of the retinoic acid receptor A (*RARA*) gene most commonly with the *PML* gene. The resulting fusion protein transcription factor has enhanced co-repressor-binding properties, increasing HDAC and DNA methyltransferase recruitment. This aberrant retinoid signalling results in potent transcriptional silencing of target genes. All *trans*-retinoic acid (ATRA), alone or combined with chemotherapy, is effective in reversing this silencing but resistance may occur. Addition of an HDI to this combination is logical in view of the enhanced co-repressor binding and HDAC recruitment by RARA fusion proteins. Histone deacetylase inhibitor therapy alone does not induce differentiation in APL but can induce this in retinoic acid-resistant cell lines when the two agents are combined (Altucci *et al*, 2005) In APL, this approach requires clinical testing to prove its effectiveness over retinoic acid treatment alone. In acute myeloid leukaemia (AML) or myelodysplastic syndrome (MDS) either unsuitable for or relapsed following conventional therapy, combined VPA and ATRA has produced modest efficacy in phase I/II clinical trials with apparently manageable toxicity. It remains unclear, from these studies, to what extent the combination adds to either drug given as monotherapy, and further data in this area would be of interest (Bug *et al*, 2005; Kuendgen *et al*, 2005, 2006).

Histone deacetylase inhibitors may be of value in combination with hormonal therapy for breast cancer. They potentiate the antiproliferative effects of the selective oestrogen receptor (ER) modulators tamoxifen and raloxifene, the pure antioestrogen fulvestrant and the aromatase inhibitor letrozole in breast cancer cell lines. Interestingly, the partial agonistic effect of endometrial adenocarcinoma cell proliferation induced by tamoxifen was blocked by HDI co-administration (Hodges-Gallagher *et al*, 2007). In a further work, treatment with an HDI rendered formerly unresponsive ER*α*-negative breast cancer cells responsive to tamoxifen. HDI enhanced overall ER transcriptional activity in these cells. Interestingly, this appeared to be by inducing the expression and nuclear translocation of ER*β* but not ER*α*. Reduction of ER*β* expression by short interfering RNA abrogated this HDI-induced sensitisation effect (Jang *et al*, 2004). Evidence exists to suggest that DNA methylation and histone deacetylation interact to maintain a repressive chromatin complex at the *ER* promoter. Inhibition of either may be sufficient to activate the silenced *ER* gene (Zhou *et al*, 2007). Clinical investigation following these observations is in progress.

## HDI combination with targeted agents for BCR/ABL-positive leukaemia

The role of HDIs in combination with agents targeting the BCR/ABL oncoprotein has been investigated in chronic myeloid leukaemia (CML) for which the BCR/ABL tyrosine kinase inhibitor imatinib is now a standard therapy but resistance can develop. *In vitro*, vorinostat has been shown to synergistically enhance the activity of imatinib and the second-generation agent dasatinib possibly through the inhibition of HSP90 chaperone function. In addition to downregulation of BCR/ABL expression, multiple perturbations in signalling and cell cycle-regulatory proteins are induced by this combination including the Ras/Raf/MEK/ERK, Akt, STAT and JNK pathways and cyclin D1 (Yu *et al*, 2003). Subsequently, combination of HDI with sorafenib, an inhibitor of multiple kinases including Raf-1, platelet-derived growth factor, vascular endothelial growth factor receptors 1 and 2, and FLT3, was found to induce synergistic cell death in BCR/ABL-positive cells, imatinib-resistant cells and primary CD34+ bone marrow cells from CML patients (Dasmahapatra *et al*, 2007).

Interestingly, both imatinib and sorafenib blocked the HDI-mediated induction of p21^WAF1/CIP1^, perhaps the most consistently observed HDI downstream molecular effect. Forced expression of p21^WAF1/CIP1^ depleted the combined HDI/sorafenib effect, implying that this may be required for synergism. Potential mechanisms include disruption of p21^WAF1/CIP1^-mediated G1 arrest, interference with its direct antiapoptotic actions such as inhibition of caspase-3 or c-Jun NH2-terminal kinase activation or disruption of the upstream Raf/MEK/ERK axis (Yu *et al*, 2003; Dasmahapatra *et al*, 2007).

## HDIs in combination with other epigenetic modifiers

One reason for the relatively modest number of genes affected by HDIs is the dominant effect of methylation status over acetylation. Dual administration of HDIs with DNA-hypomethylating agents is therefore of interest. Aberrant DNA methylation is characteristic of a number of myeloid leukaemias (Garcia-Manero *et al*, 2002) and dual epigenetic modulation might allow suppression of the malignant clone. *In vitro*, the combination of VPA and 5-aza-2′-deoxycytidine (decitabine) produced synergistic growth inhibition and induction of apoptosis in leukaemia cell lines (Yang *et al*, 2005). In a subsequent phase I/II clinical trial of this combination, 54 patients with AML or high-risk MDS, either relapsed or unsuitable for first-line chemotherapy, received a fixed dose of decitabine and escalating doses of VPA. Twelve patients (22%) had objective responses with 10 complete remissions. Major cytogenetic response was documented in six of eight responders. Of the five target genes investigated, hypomethylation of the key cell cycle-regulating gene, *p15* was found to be the best indicator of response. Pretreatment *p15* methylation was significantly lower in responders *vs* non-responders; however, neither the absolute or the percentage change in *p15* methylation was statistically significant, and responses were not correlated with the induction of H3 or H4 acetylation (Garcia-Manero *et al*, 2006). In another study, utilising phenylbutyrate and 5-azacytidine in a similar patient population, 11 of 29 patients responded. Furthermore, six of six with pretreatment methylation of *p15* or *CDH-1* (E-cadherin) promoters reversed methylation during the first cycle of therapy whereas none of the six non-responders showed any demethylation (Gore *et al*, 2006). Further identification and validation of predictive biomarkers would be of immense value in the ongoing clinical assessment of HDIs in this and other settings but may need to be tumour- and combination-specific. Toxicity in these clinical studies included neurological (encephalopathy, confusion, somnolence) and haematological (thrombocytopaenia, neutropaenia) events and fatigue, but the combinations were considered well tolerated. Further clinical evaluation will be required to establish the value of combination therapy over monotherapy approaches and compared to conventional chemotherapeutic interventions.

## HDI combination with ionising radiation

Histone deacetylase inhibitors can act as radiosensitisers (Karagiannis and El-Osta, 2006). For example, in NSCLC cells, synergism has been demonstrated between HDIs and irradiation for induction of apoptosis and inhibition of clonogenic survival and confirmed in *in vivo* tumour studies. Following irradiation, *γ*-H2AX foci following irradiation, a conserved response to DNA double-strand break formation necessary for recruitment of many factors involved in DNA repair was found to be increased by combination with an HDI. Furthermore, radiation alone induced translocation of HDAC4 to the nucleus whereas combination therapy resulted in its confinement to the cytoplasm (Geng *et al*, 2006). The DNA damage-sensing protein, 53BP1, has been shown to co-localise to the nucleus with HDAC4 in response to double-strand DNA breaks (Kao *et al*, 2003). Therefore, HDI therapy may potentiate radiation in part by the suppression of HDAC incorporation into DNA damage-signalling and -repair complexes. Other factors are also likely to be relevant. For example, following DNA damage, checkpoint molecules activate ataxia telangiectasia-mutated protein (ATM), which in turn phosphorylates effectors. Histone deacetylase-1 is known to interact with ATM and this interaction is enhanced by ionising radiation and inhibited by HDAC inhibition (Kim *et al*, 1999). Histone deacetylases are also important for the repair of established double-strand breaks, with the expression of Ku70, Ku86 and other repair proteins decreased by HDI therapy despite radiation-induced DNA damage (Munshi *et al*, 2005). These pre-clinical studies demonstrate the range of potential mechanisms that are implicated in HDI-mediated radiosensitisation and a variety of clinical studies are in progress (Table 1).

## Conclusions

The niche for HDIs in the treatment of cancer remains inadequately defined. Understanding of the diverse mechanisms for their anticancer action continues to increase, including molecular mechanisms elucidated by studying combination therapy. In the face of modest activity as single agents, except in cutaneous T-cell lymphoma (CTCL) where the unique tumour microenvironment may account for their unpredicted efficacy, their ability to synergise with, and potentially overcome resistance to, many other agents represents a promising strategy for clinical development. Current evidence to support this assertion is predominantly pre-clinical, with only a small number of non-randomised early-phase clinical trials reported (of combinations with anthracyclines, ATRA or DNA-hypomethylating agents). We therefore wait for clear proof that the multiple promising combinations tested in pre-clinical studies can in fact translate to added clinical value for patients above use of single or other agents. If this can be achieved, then the next few years should herald clinical data from phase II and III studies to define the exact value of combination approaches and improved understanding of mechanisms that underpin activity.

## Figures and Tables

**Table 1:** A search of the NCI clinical trial database (www.cancer.gov/clinicaltrials) was conducted on 20 December 2007 (protocol search id 3988767) to identify all clinical trials of HDIs in combination with other agents currently recruiting patients

				**Agents under investigation in combination with HDI**	
**Compound**	**Class**	**Potency**	**Tumour site/type**	**Phase I clinical trials**	**Phase II clinical trials**
SAHA (Vorinostat), Merck (Whitehouse Station, NJ, USA)	Hydroxamate	∼*μ*M	MDS; MM; AML; renal; NSCLC; colorectal; GBM; upper gastrointestinal; ovarian; breast	Bortezomib; bexarotene; 5-flourouracil, leucovorin and oxaliplatin; isotretinoin; gemcitabine; temozolamide; decitabine; doxorubicin; idarubicin; flavopiridol; 5-azacitidine; cytarabine and etoposide; irinotecan, 5-flourouracil and leucovorin; docetaxel	Bevacizumab; isotretinoin; 5-flourouracil; paclitaxel and bevacizumab; bortezomib; decitabine; erlotinib; isotretinoin and carboplatin; tamoxifen; carboplatin and paclitaxel, 5-azacitidine
PXD101 (Belinostat), CuraGen (Branford, CT, USA)	Hydroxamate	∼*μ*M	Lymphoma; advanced haematological malignancy; solid tumours; ovarian	Bortezomib; isotretinoin; 5-azacytidine; 5-flourouracil	Bortezomib, carboplatin and paclitaxel
LBH589, Novartis (Basel, Switzerland)	Hydroxamate	∼nM	MM; breast; solid tumours	Ketoconazole; bortezomib; lenalidomide and dexamethasone; paclitaxel, carboplatin and bevacizumab	Trastuzumab, gemcitabine
FK228 (Romidepsin), Gloucester Pharmaceuticals (Cambridge, MA, USA)	Cyclic peptide	∼nM	MM; pancreatic; lung and pleural	Decitabine; flavopiridol	Bortezomib; gemcitabine
Valproic Acid	Aliphatic acid	∼mM	AML; CLL; small lymphocytic lymphoma; aggressive B-cell NHL; MDS; melanoma; ovarian; GBM; breast	Decitabine; etoposide; bevacizumab	Karenitecin; 5-azacytidine and ATRA; carboplatin and 5-azacytidine; temozolamide and external beam radiotherapy; 5-flourouracil, epirubicin and cyclophosphamide
MGCD0103, Methylgene (Montreal, QC, Canada)	Benzamide	∼*μ*M	MDS; AML; solid tumours	Docetaxel	5-azacitidine, gemcitabine

AML=acute myeloid leukaemia; ATRA=all-*trans* retinoic acid; CLL=chronic lymphocytic leukaemia; GBM=glioblastoma multiforme; HDI=histone deacetylase inhibitor; MDS=myelodysplastic syndrome; MM=multiple myeloma; NHL=non-Hodgkin's lymphoma; NSCLC=non-small cell lung cancer.
